# Nonalcoholic fatty liver disease and the risk of insulin-requiring gestational diabetes

**DOI:** 10.1186/s13098-021-00710-y

**Published:** 2021-08-26

**Authors:** Sang Youn You, Kyungdo Han, Seung-Hawn Lee, Mee Kyoung Kim

**Affiliations:** 1 College of Medicine, The Catholic University of Korea, Seoul, 06591 South Korea; 2 Department of Statistics and Actuarial Science, Soongsil University, Seoul, 06978 South Korea; 3 Division of Endocrinology and Metabolism, Department of Internal Medicine, Seoul St. Mary’s Hospital, College of Medicine, The Catholic University of Korea, #222 Banpo-daero, Seocho-gu, Seoul, 06591 South Korea; 4 Department of Medical Informatics, College of Medicine, The Catholic University of Korea, Seoul, 06591 South Korea; 5 Division of Endocrinology and Metabolism, Department of Internal Medicine, College of Medicine, Yeouido St. Mary’s Hospital, The Catholic University of Korea, #10 63-ro, Yeongdeungpo-gu, Seoul, 07345 South Korea

## Abstract

**Background:**

Nonalcoholic fatty liver disease (NAFLD) is one of the most common chronic liver diseases; however, there has been little research into its impact on gestational diabetes mellitus (GDM).

**Methods:**

This study included 308,095 women registered in the Korean National Health Insurance Service database, who delivered between 2011 and 2015 and received a health examination within 52 weeks before pregnancy. Insulin-requiring GDM was defined as no insurance claims for diabetes mellitus and a fasting blood glucose level of < 126 mg/dL before pregnancy, and initiation of insulin treatment during pregnancy. A fatty liver index (FLI) was calculated using body mass index, waist circumference, and blood triglyceride and γ-glutamyl transferase levels. FLI scores < 30 ruled out hepatic steatosis, while FLI scores ≥ 60 indicated NAFLD.

**Results:**

The prevalence of NAFLD was 0.8% (2355/308,095) and 1984 (0.6%) subjects developed insulin-requiring GDM. FLIs of 30–59 and ≥ 60 were significantly associated with increased risk of insulin-requiring GDM (odds ratio [OR] 3.50; 95% confidence interval [CI] 2.99–4.10; OR 4.19; 95% CI 3.37–5.23), respectively. Further exploration of the association of FLI with GDM across FLI decile categories revealed a steady increase in OR across the categories. The association was more prominent among those without metabolic syndrome.

**Conclusion:**

NAFLD in women is an independent risk factor for insulin-requiring GDM.

**Supplementary Information:**

The online version contains supplementary material available at 10.1186/s13098-021-00710-y.

## Introduction

Nonalcoholic fatty liver disease (NAFLD) is defined as an increase in liver fat content, in the absence of any secondary cause of steatosis [1, 2]. The prevalence of NAFLD increases in parallel with the increasing prevalence in obesity, metabolic syndrome (MetS), and type 2 diabetes mellitus (DM) [1, 2]. Many longitudinal studies have shown that NAFLD is an independent risk factor for developing type 2 DM [3, 4]. These different metabolic diseases, NAFLD and type 2 DM, share a common metabolic dysfunction of insulin resistance. The insulin-resistant fatty liver overproduces glucose and very-low-density lipoprotein [5]. This boosts mechanisms that lead to exhaustion of the pancreatic beta cell reserve, eventually leading to the development of DM [5]. Steatotic and inflamed liver secretes hepatokines such as fetuin-A, fetuin-B, angiopoietin-like proteins, fibroblast growth factor 21, and selenoprotein P, which have endocrine functions at extrahepatic sites to cause insulin resistance and other adverse effects on glucose homeostasis [6]. Previous studies have shown an association between a history of gestational diabetes mellitus (GDM) and NAFLD in women [7, 8]. We hypothesized that NAFLD before pregnancy could be a risk factor for the development of GDM.

GDM is a common international health problem in pregnant women, which can lead to adverse pregnancy outcomes [9]. As the number of women being diagnosed with GDM has increased in past decades, efforts are increasing to identify risk factors for GDM [9]. The severity of GDM is associated with maternal blood glucose levels that present a direct correlation with the risk of fetal involvement [10]. A need for insulin therapy might be a starting point for the characterization of patients with severe GDM related to greater difficulty in achieving glycemic control [10, 11]. It is important to identify subjects who are at risk of developing severe GDM. Therefore, we conducted a large population-based study involving more than 300,000 pregnant women in Korea who received a health examination within 52 weeks before pregnancy to examine the prognostic significance of NAFLD before pregnancy for the risk of severe GDM.

## Methods

### Data source and study population

Using the Korean National Health Insurance Service (NHIS) database, we retrospectively recruited pregnant women from the population for the current study. The NHIS is managed by the government and is the sole insurer for health-care services with a coverage rate of approximately 97% of the population in the Republic of Korea. The NHIS database is available for population-based cohort studies. Information on demographics, national health screening data, diagnosis statements defined by the International Classification of Disease 10^th^ revision (ICD-10) codes, medical treatments, and drug prescriptions is routinely collected and undergoes quality control before being released for research purposes [12–14]. Enrollees in the NHIS are recommended to undergo a standardized medical examination at least every 2 years. This regular health examination includes anthropometric measurements, assessment of blood pressure, alcohol and smoking status, and physical activity in addition to laboratory tests after overnight fasting for serum glucose, total cholesterol, triglycerides, creatinine, liver function, and urinalysis. In this study, we searched the NHIS database to identify women who had delivered between 2011 and 2015 and estimated the date of conception as 280 days before the delivery date (Additional file 1: Figures S1, S2). Women who had undergone a health examination within 52 weeks before conception were selected (n = 329,675). We excluded women who had DM before pregnancy (n = 2303) and had fasting blood glucose levels ≥ 126 mg/dL at the health examination (n = 1278) or with missing data for at least one variable (n = 4570). Women with excessive alcohol use (≥ 30 g/day, n = 6928) and a history of viral hepatitis, autoimmune hepatitis or other forms of chronic liver disease (n = 6501) were also excluded from the analysis. Finally, 308,095 women were included in this study, which was approved by the Institutional Review Board of Seoul St. Mary’s Hospital, Seoul, The Catholic University of Korea (No. KC19ZESI0586). Informed consent was waived because we used deidentified and anonymous information in this study.

### Calculation of the fatty liver index

We calculated the fatty liver index (FLI) according to the formula below, which incorporated levels of triglycerides and γ-glutamyl transferase (GGT), and body mass index (BMI) and waist circumference (WC) [15, 16]:

$$\text{FLI }=(\text{exp }[\text{Mode}{{\text{l}}_{\text{FLI}}}\left] )/(\text{1 }+\text{ exp } \right[\text{Mode}{{\text{l}}_{\text{FLI}}}])\times 100$$

$$\text{Mode}{{\text{l}}_{\text{FLI}}}=\text{ }(0.0\text{953}\times \text{ln }\left( \text{triglyceride }\left[ \text{mg}/\text{dl} \right] \right)\text{ }+\text{ }(0.\text{139}\times \text{BMI }\left[ \text{kg}/{{\text{m}}^{\text{2}}} \right])\text{ }+\text{ }(0.\text{718}\times \text{ln GGT }\left[ \text{IU}/\text{l} \right])\text{ }+\text{ }(0.053\times \text{WC }\left[ \text{cm} \right])\text{ } 15.745.$$

We classified the study population into three groups according to the FLI as follows [13, 14]: low-risk group, defined as FLI < 30; intermediate-risk group, defined as 30 ≤ FLI < 60; and high-risk group, defined as FLI ≥ 60. FLI scores < 30 ruled out hepatic steatosis, while FLI ≥ 60 indicated NAFLD [15, 16].

### Measurements and definitions

We defined obesity as a BMI ≥ 25.0 kg/m^2^, according to the World Health Organization Western Pacific Region guideline [17]. Abdominal obesity was defined as a WC ≥ 85 cm [18]. Information on smoking status was obtained from a self-reported health survey questionnaire (current smoker, defined as those who had smoked over 5 packs during their lifetime and continued to smoke). Drinking status was defined as mild (< 30 g/day) or nondrinking. Regular exercise was defined as moderate physical activity performed for more than 20 min at least 3 times per week or strenuous physical activity performed more than 30 min at least 5 times per week. We dichotomized household income levels at the lowest 25% for the analysis.

Insulin-requiring GDM was defined as having no history of previous diabetes and receiving a prescription for insulin during the pregnancy. Participants with non-GDM or GDM without insulin treatment were treated as the control group.

### Statistical analysis

Continuous variables are presented as mean ± standard deviation (SD) and median (25–75%), while the categorical variables are presented as n (%) for each group. The participants were classified into three groups according to cut-off scores of FLI (30 and 60). We performed one-way analyses of variance (ANOVA) or Chi-square test, as appropriate to compare each group. Multiple logistic regression analysis was performed to obtain odds ratios (ORs) and 95% confidence intervals (CIs) for GDM. The multivariable-adjusted models used in the analysis were as follows: model 1 was adjusted for age; model 2 was adjusted further for socioeconomic status (smoking, alcohol drinking, regular exercise, and income status), fasting blood glucose, and dyslipidemia; and model 3 was adjusted further for family history of diabetes. The potential modification effect caused by age, smoking, hypertension, dyslipidemia, and MetS was identified through a stratified analysis and interaction testing using the likelihood-ratio test. We also performed the same analyses according to the FLI components (BMI, WC, triglycerides, and GGT highest quartile). SAS software (version 9.4; SAS Institute, Cary, NC, USA) was used for the analyses and a *P* value < 0.05 was considered to indicate statistical significance.

## Results

### Clinical characteristics of the study population before pregnancy

The prepregnancy characteristics of the study population according to their baseline FLI categories are shown in Table 1. In this population, 2355 subjects (0.8%) were identified as having NAFLD (FLI scores ≥ 60), while 7265 subjects (8.7%) had a FLI score of 30–59. Subjects with FLI scores ≥ 60 were older, more likely to be current smokers and had a lower income (lower 25%) than subjects with a FLI score < 30. Subjects with FLI scores ≥ 60 were more obese and had a higher prevalence of MetS.

**Table 1:** Clinical characteristics of the study subjects before pregnancy according to fatty liver index score category

	Fatty liver index score		
	< 30	30–59	≥ 60
N	298,475	7265	2355
Age (years)	29.6 ± 3.6	31.1 ± 4.4	31.3 ± 4.2
≥ 35 years	25,232 (8.5)	1370 (18.9)	467 (19.8)
Current smoker	9867 (3.3)	688 (9.5)	319 (13.6)
Mild alcohol drinker	144,475 (48.4)	3986 (54.9)	1312 (55.7)
Regular Exercise	31,079 (10.41)	932 (12.83)	303 (12.87)
Income (lower 25%)	58,363 (19.55)	2131 (29.33)	781 (33.16)
Family history of DM	28,016 (13.0)	1060 (20.3)	345 (19.9)
BMI (kg/m^2^)	20.5 ± 2.3	27.5 ± 2.8	31.5 ± 3.6
< 18.5	52,986 (17.8)	3 (0.04)	1 (0.04)
18.5–22.9	204,030 (68.4)	378 (5.2)	21 (0. 9)
23–24.9	27,633 (9.3)	963 (13.3)	39 (1.7)
25–29.9	13,498 (4.5)	4528 (62.3)	718 (30.5)
≥ 30	328 (0.1)	1393 (19.2)	1576 (66.9)
Waist circumferences (cm)	68.7 ± 6.2	84.9 ± 6.5	93.8 ± 8.0
Triglyceride (mg/dL)	66.9 (66.8–67.0)	137.8 (136.4–139.3)	172.6 (169.3–176.0)
GGT (IU/L)	15.1 (15.0–15.1)	28.7 (28.4–29.1)	40.3 (39.4–41.6)
FBG (mg/dL)	87.1 ± 9.0	92.3 ± 10.5	94.7 ± 11.3
TC (mg/dL)	176.32 ± 28.33	197.88 ± 33.73	205.89 ± 35.29
AST (IU/L)	14.0 (14.0–14.1)	23.3 (23.0–23.6)	32.4 (31.6–33.2)
ALT (IU/L)	19.2 (19.2–19.3)	22.6 (22.4–22.7)	27.1 (26.6–27.5)
Systolic BP (mmHg)	109.64 ± 10.55	118.68 ± 11.89	124.09 ± 12.91
Diastolic BP (mmHg)	68.97 ± 7.94	74.91 ± 8.77	78.7 ± 9.5
Hypertension (yes)	3102 (1.0)	524 (7.2)	349 (14.8)
Metabolic syndrome (yes)	1739 (0.6)	1691 (23.3)	1374 (58.3)

Data are expressed as the mean ± SD, median (25–75%), or n (%). P-values for the trend were < 0.0001 for all variables because of the large size of the study population

AST: aspartate transaminase; ALT: alanine transaminase; BMI: body mass index; BP: blood pressure; DM: diabetes mellitus; FBG: fasting blood glucose; GGT: gamma-glutamyl transferase; TC: total cholesterol

### Risk of insulin-requiring GDM according to the FLI

There were 1,984 (0.6%) women with GDM who received insulin therapy. Compared to subjects with FLI scores < 30, the age-adjusted ORs for subjects with FLI 30–59 and FLI ≥ 60 were 5.70 (95% CI 4.98–6.53) and 9.96 (95% CI 8.35–11.90) for insulin-requiring GDM, respectively (Table 2). These associations persisted after further adjustment for smoking, alcohol drinking, regular exercise, income status, fasting blood glucose, and dyslipidemia (model 2). The multivariable-adjusted ORs for subjects with FLI scores 30–59 and ≥ 60 were 3.53 (95% CI 3.06–4.07) and 4.83 (95% CI 3.99–5.84) for insulin-requiring GDM, respectively.

**Table 2:** Incidence rate and adjusted odd ratios (95% confidence intervals) for the risk of insulin-requiring gestational diabetes by fatty liver index and each component of fatty liver index

	Events (n)	Incidence rate*	Model 1	Model 2	Model 3
Fatty liver index					
< 30	1570	5.3	1 (Ref.)	1 (Ref.)	1 (Ref.)
30–59	267	36.8	5.70 (4.98,6.53)	3.53 (3.06,4.07)	3.50 (2.99,4.10)
≥ 60	147	62.4	9.96 (8.35,11.90)	4.83 (3.99,5.84)	4.19 (3.37,5.23)
Body mass index ≥ 25 kg/m^2^					
No	1413	4.9	1 (Ref.)	1 (Ref.)	1 (Ref.)
Yes	571	25.9	4.52 (4.09,5.00)	3.14 (2.83,3.49)	3.01 (2.67,3.39)
Waist circumference ≥ 85 cm					
No	1676	5.6	1 (Ref.)	1 (Ref.)	1 (Ref.)
Yes	308	29.6	4.45 (3.92,5.04)	2.89 (2.54,3.29)	2.85 (2.46,3.30)
Triglyceride ≥ 150 mg/dl or lipid lowering treatment					
No	1584	5.4	1 (Ref.)	1 (Ref.)	1 (Ref.)
Yes	400	27.6	4.30 (3.84,4.81)	2.82 (2.50,3.18)	2.79 (2.44,3.20)
Gamma-glutamyl transferase (GGT) highest quartile, ≥ 18 IU/L					
No	1007	4.3	1 (Ref.)	1 (Ref.)	1 (Ref.)
Yes	977	12.9	2.78 (2.54,3.04)	2.19 (2.00,2.40)	2.22 (2.00,2.47)

*Per 1000 person-years

Model 1: Adjusted for age

Model 2: Adjusted for age, smoking, alcohol drinking, regular exercise, income status, fasting blood glucose, and dyslipidemia

Model 3: Adjusted for model 2 + family history of diabetes

**Fig. 1:**
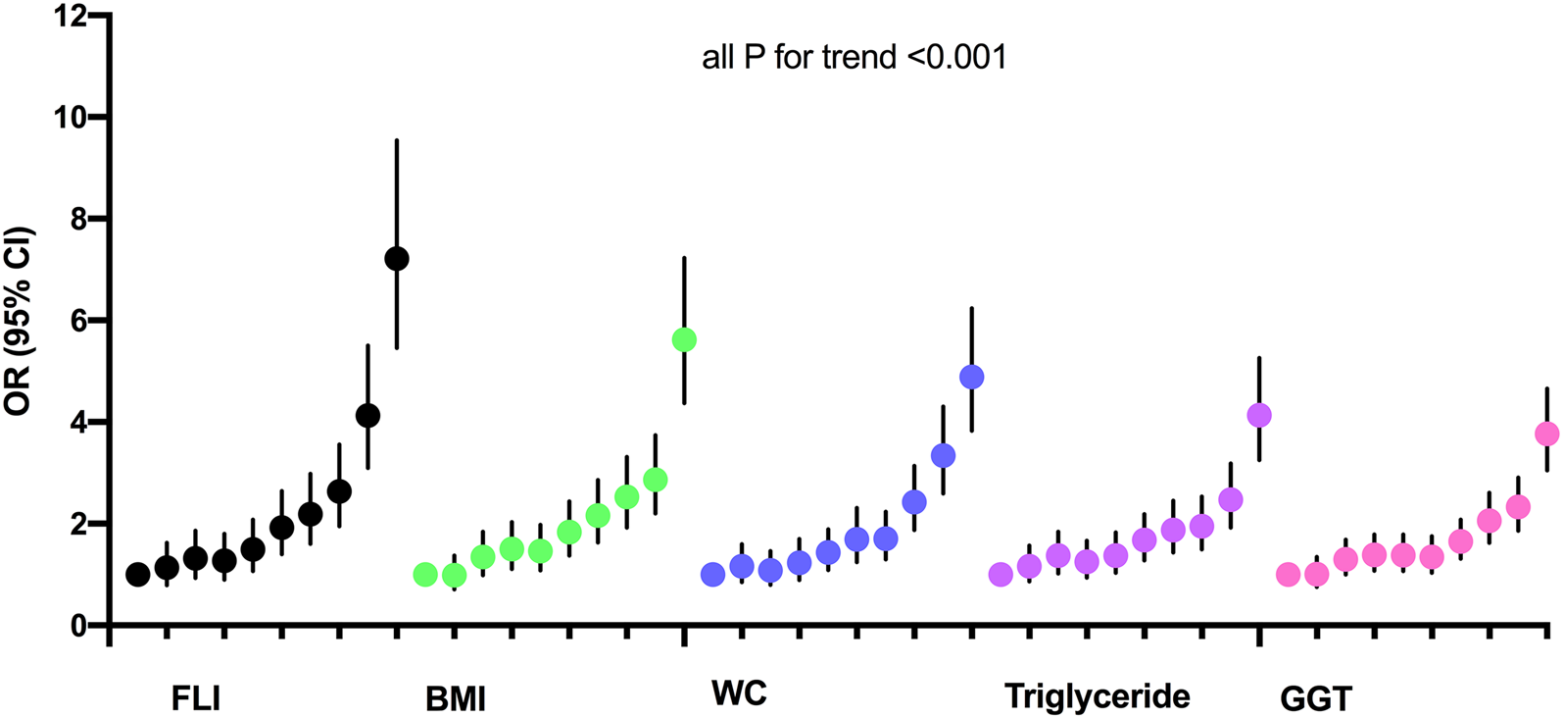
Adjusted odd ratios and 95% confidence intervals of insulin-requiring gestational diabetes by deciles of fatty liver index score and deciles of each component of the fatty liver index. Adjusted for age, smoking, alcohol drinking, regular exercise, income status, fasting blood glucose, and dyslipidemia

Further exploration of the association of FLI with GDM across FLI decile categories revealed a steady increase in OR across the categories (Fig. 1). Women within the 10th decile of FLI scores (D10 > 12.5) were at greatest risk with a 622% increase in risk (OR 7.22; 95% CI 5.46–9.54), compared with those with FLI scores in D1 (D1 < 1.3).

Individual components of the FLI were also associated with the risk of insulin-requiring GDM (Table 2, Fig. 1). However, these ORs were not stronger than the ORs between FLI and GDM (Fig. 1).

### Subgroup analyses by age, smoking, hypertension, dyslipidemia, and MetS

We performed subgroup analyses according to age, smoking, absence or presence of hypertension, dyslipidemia, and MetS (Table 3). In all subgroups, the ORs of insulin-requiring GDM displayed an increasing trend as the FLI category increased. However, two subgroup analyses according to age and MetS displayed significant differences in ORs of insulin-requiring GDM (*P* values for interaction were 0.03 and < 0.001, respectively). Higher adjusted ORs for insulin-requiring GDM were observed in the younger-aged (age < 35 years) and non-MetS groups. When compared with subjects having FLI scores < 30 and without MetS, having a FLI score ≥ 60 without MetS was associated with a 3.5-fold increased risk of insulin-requiring GDM (OR 3.50; 95% CI 2.41–5.09). Compared with subjects with a FLI score < 30 and MetS, having a FLI ≥ 60 and MetS was associated with a 2.1-fold increased risk of insulin-requiring GDM (OR 2.09; 95% CI 1.53–2.86). These findings suggest that the utility of the FLI as a risk factor for GDM may be more valid in these subpopulations.

**Table 3:** Adjusted odd ratios (95% confidence intervals) of insulin-requiring gestational diabetes by fatty liver index score category in subgroups according to age, smoking, hypertension, dyslipidemia, and metabolic syndrome

	Subgroup	Fatty liver index			P for interaction
		< 30	30–59	≥ 60	
Age	< 35	1 (Ref.)	3.85 (3.26,4.56)	5.06 (4.02,6.38)	0.03
	≥ 35	1 (Ref.)	2.92 (2.26,3.78)	4.28 (3.06,5.99)	
Smoker	No	1 (Ref.)	3.59 (3.08,4.17)	4.76 (3.86,5.86)	0.78
	Yes	1 (Ref.)	3.23 (2.13,4.90)	5.19 (3.19,8.43)	
Hypertension	No	1 (Ref.)	3.53 (3.04,4.09)	4.83 (3.93,5.94)	0.72
	Yes	1 (Ref.)	3.16 (1.78,5.58)	4.49 (2.50,8.08)	
Dyslipidemia	No	1 (Ref.)	3.38 (2.89,3.94)	5.20 (4.22,6.41)	0.14
	Yes	1 (Ref.)	4.31 (2.99,6.20)	3.95 (2.52,6.20)	
Metabolic syndrome	No	1 (Ref.)	3.19 (2.67,3.81)	3.50 (2.41,5.09)	< 0.001
	Yes	1 (Ref.)	1.62 (1.19,2.19)	2.09 (1.53,2.86)	

Adjusted for age, smoking, alcohol drinking, regular exercise, income status, fasting blood glucose, and dyslipidemia

In additional sensitivity analyses, with the combination of FLI category and MetS status as a composite exposure variable, the risk of GDM was compared to subjects having FLI scores < 30 and without MetS (reference group). Women having a FLI score ≥ 60 and MetS were at the greatest risk of insulin-requiring GDM (Additional file 1: Figure S3).

## Discussion

In this study, we demonstrated that the presence of NAFLD before pregnancy was associated with an increased risk of insulin-requiring GDM. We identified a stronger association among women without MetS before pregnancy, which supports the hypothesis that NAFLD is an independent risk factor for GDM, regardless of MetS status.

Our results are consistent with the findings of previous studies that demonstrated an association between NAFLD and GDM. A recent cohort study in a population of Korean pregnant women who visited two hospitals in Korea for prenatal care investigated whether the presence of NAFLD in the first trimester was a risk factor for GDM in mid-gestation [19]. In this population, 5.3% of all subjects had FLI scores ≥ 60 [19], while in our study, only 0.8% had FLI scores ≥ 60. The previous study’s participants were composed of individuals who visited the two hospitals (secondary-level or university hospital) for prenatal care before 14 weeks of gestation [19]. Pregnant women attending secondary-level or university hospitals may have more risk factors than women attending primary hospitals. The differences in the prevalence of NAFLD appear to be related to differences in the population studied and different timing of NAFLD assessment (10–14 weeks of gestation vs. pre-pregnancy). Our study enrolled a large population representing > 300,000 deliveries. Because of the national health insurance coverage, almost all pregnant women in Korea undergo GDM screening and treatment during pregnancy; therefore, our findings reflect ‘real-world’ data, on a national scale, regarding the impact of pre-pregnancy NAFLD on the risk of GDM in Korean women.

Previous studies of the association between NAFLD and the risk of GDM did not consider the MetS status of the subjects [7, 19, 20]. However, a number of studies have looked into the association of GDM and components of the MetS [20, 21]. Hagström et al. [20] reported that the effect of NAFLD on the risks of preeclampsia and GDM was primarily seen in women with a BMI < 30 kg/m^2^. NAFLD did not influence any adverse outcomes of pregnancy among women with a BMI of ≥ 30 [20]. A recent study demonstrated that a high FLI category is associated with an increased risk of the incidence of type 2 DM in men without MetS [21]. In our study, we also identified that the association was stronger among women without MetS before pregnancy, suggesting that among women with MetS, which represents a cluster of risk factors for diabetes, a high FLI has less impact on the risk of GDM.

We discovered that the FLI was associated with risk of GDM in a dose-dependent manner. Comparison of risk using the FLI 1st decile as a reference revealed a steady increase in risk across FLI categories. The FLI is a multivariate model used to estimate fat accumulation in the liver and has been validated in multiple model systems [22]. Given that sonographic estimation of fatty liver is largely subjective and examiner-dependent, the observation that the FLI is associated with the risk of GDM reinforces our understanding of the clinical significance of NAFLD in pregnancy. The optimal cut-off point of the FLI for diagnosing NAFLD was 30 in middle-aged Chinese subjects [23]. In our study population, the 10th decile range of FLI was above 12.5. According to a study conducted in Taiwan, for men, the optimal cut-off scores are an FLI < 25 to rule out and FLI ≥ 35 to rule in sonographic fatty liver [24]. For women, a FLI < 10 for exclusion and FLI ≥ 20 for inclusion of fatty liver were nominated [24]. Due to variations in ethnicity, and dietary and environmental factors, the cut-off for WC and BMI is different for Asian people. Therefore, the FLI needs to be validated when used in different populations and cut off values for the FLI (30 and 60) should be rebalanced for appropriate application in women of childbearing age.

The current study had some limitations that warrant discussion. First, we only used the FLI to diagnose NAFLD and did not use histological examination and/or liver ultrasound. Liver biopsy is the gold standard for diagnosis and staging of NAFLD, but it cannot be applied to population-based studies because of its highly invasiveness. Liver ultrasonography is not included in national health screening due to the lack of cost-effectiveness of mass screening.

Furthermore, liver ultrasonography is known to be a weak diagnostic tool for NAFLD, especially in the lower range (< 10 ~ 15%) of hepatic steatosis [25]. Second, there is currently little research to verify the validity of the FLI in the Korean population. One study reported that the area under the receiver–operator characteristic curve of FLI in Korean subjects is 0.86, which is a relatively high level [16]. Third, we did not have data on transient elastography (TE) or acoustic radiation force impulse (ARFI). TE or ARFI data to evaluate liver fibrosis in patients with NAFLD could be helpful in understanding the correlation between the degree of fibrosis or severity of steatosis and the development of GDM. It was recently reported that elevated gamma-glutamyl transferase (≥ 18 U/L), and alanine aminotransferase (≥ 17 U/L) or elevation of both liver enzyme levels before pregnancy were independent risk factors for GDM in a subsequent pregnancy [26]. Fourth, we did not study data on insulin resistance such as HOMA-IR (Homeostatic Model Assessment for Insulin Resistance), since it is difficult to conduct these tests for all participants in a mass screening program. Finally, the current study consisted of a Korean population only; therefore, these findings may not be able to be generalized to other ethnicities.

In the current study, the OR (95% CI) of NAFLD for GDM was 4.83 (3.99, 5.84), implying that NAFLD should be considered a major risk factor for the development of GDM. We found a stronger association among women without MetS before pregnancy, which supports the hypothesis that NAFLD is an independent risk factor for GDM, regardless of MetS status. Early identification of women with NAFLD is important and more intensive screening and preventive strategies are needed for this subpopulation.

## Supplementary Information

**Additional file 1: Figure S1.** Flowchart of the study population. **Figure S2.** Timeline for the Study data collection. **Figure S3.** Adjusted odd ratios (95% confidence intervals) of insulin-requiring gestational diabetes according to the presence of metabolic syndrome (MetS) and fatty liver index (FLI) category. Subjects with a FLI <30 and no MetS were analyzed as a reference group. Adjusted for age, smoking, alcohol drinking, regular exercise, income status, fasting blood glucose, and dyslipidemia.

## Data Availability

The datasets used and/or analyzed in the current study are available from the corresponding author upon reasonable request.

## Abbreviations

BMI: Body mass index
FLI: Fatty liver index
GDM: Gestational diabetes mellitus
GGT: γ-Glutamyl transferase
MetS: Metabolic syndrome
NAFLD: Nonalcoholic fatty liver disease
NHIS: National Health Insurance System
WC: Waist circumference
