# Supplementary material for: Nonalcoholic fatty liver disease and the risk of insulin-requiring gestational diabetes
Source: Diabetol Metab Syndr. 2021 Aug 26;13:90. doi: 10.1186/s13098-021-00710-y (PMC8393465; doi:10.1186/s13098-021-00710-y)

**Supplemental Figure S1.** Flowchart of the study population

329,675

Women with delivery between 2011 and 2015 and who had undergone a health examination within 52 weeks before pregnancy

-2,303 subjects were excluded having diabetes mellitus before pregnancy

-1,278 subjects with fasting blood glucose ≥126 mg/dl were excluded.

-4,570 subjects were excluded with missing data on at least one variable

-6,501 subjects with a history of viral hepatitis, autoimmune hepatitis or other forms of chronic liver disease

were excluded

-6,928 subjects with excessive alcohol use were excluded

n = 308,095

Subjects eligible for inclusion

**Supplemental Figure S2.** Timeline for the Study data collection

**Delivery**

**(2011-2015)**

52weeks before conception

**Conception**

280 days

**Gestational diabetes** (yes/no)

Median time between evaluation period and delivery: 1.26±0.29 years

**Health examination**

: Assessment of NAFLD

**Supplemental Figure S3.** Adjusted odd ratios (95% confidence intervals) of insulin-requiring gestational diabetes according to the presence of metabolic syndrome (MetS) and fatty liver index (FLI) category. Subjects with a FLI <30 and no MetS were analyzed as a reference group. Adjusted for age, smoking, alcohol drinking, regular exercise, income status, fasting blood glucose, and dyslipidemia.


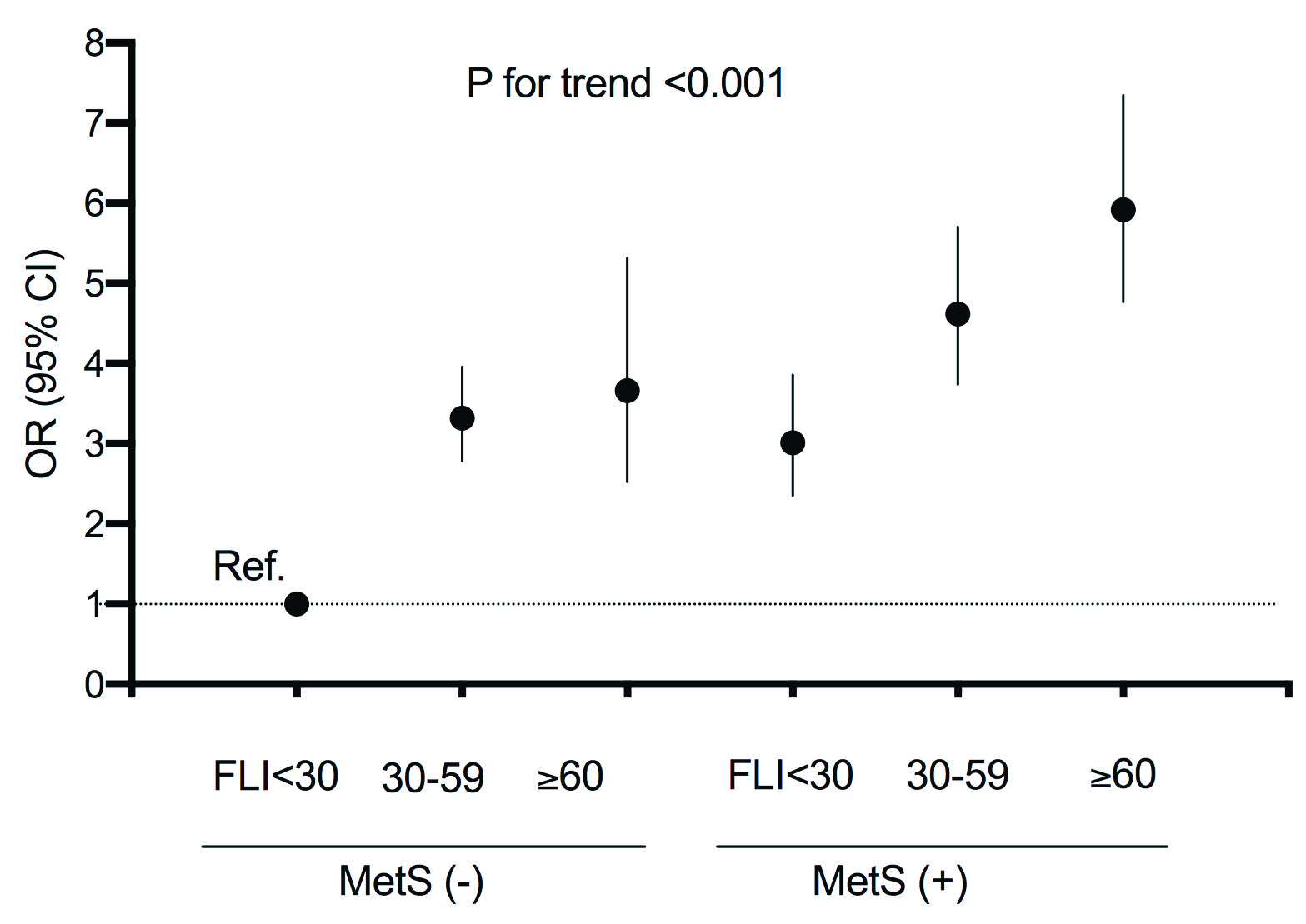

Supplement: Supplementary file 1 — Additional file 1: Figure S1. Flowchart of the study population. Figure S2. Timeline for the Study data collection. Figure S3. Adjusted odd ratios (95% confidence intervals) of insulin-requiring gestational diabetes according to the presence of metabolic syndrome (MetS) and fatty liver index (FLI) category. Subjects with a FLI <30 and no MetS were analyzed as a reference group. Adjusted for age, smoking, alcohol drinking, regular exercise, income status, fasting blood glucose, and dyslipidemia. [file 13098_2021_710_MOESM1_ESM.docx]
